# The CCR5Δ32 allele is not a major predisposing factor for severe H1N1pdm09 infection

**DOI:** 10.1186/1756-0500-7-504

**Published:** 2014-08-07

**Authors:** Manuela Sironi, Rachele Cagliani, Chiara Pontremoli, Marianna Rossi, Guglielmo Migliorino, Mario Clerici, Andrea Gori

**Affiliations:** 1 Scientific Institute IRCCS E. MEDEA, Bioinformatics, 23842 Bosisio Parini, Italy; 2 “San Gerardo” Hospital, University of Milano-Bicocca, 20900 Monza, Italy; 3 University of Milan, 20090 Milan, Italy; 4 Don C. Gnocchi Foundation ONLUS, IRCCS, 20148 Milan, Italy

**Keywords:** H1N1pdm09 infection, CCR5Δ32, Disease severity

## Abstract

**Background:**

Host genetic factors are thought to modulated the severity of disease caused by infection with the 2009 H1N1 pandemic influenza virus (H1N1pdm09). The human *CCR5* gene encodes a cytokine receptor important for cell-mediated immune response against H1N1pdm09. A 32-bp polymorphic deletion in the coding sequence of *CCR5*, the so-called CCR5Δ32 allele, segregates in populations of European ancestry with a frequency of 8-15%. A high proportion of CCR5Δ32 heterozygotes was reported in a sample of white Canadian critically-ill H1N1pdm09 infected subjects, suggesting an association with disease severity.

**Methods:**

We recruited 29 H1N1pdm09 infected subjects from Southern Europe (mostly Italians) with a wide clinical spectrum of disease symptoms; the sample included 7 subjects who developed acute respiratory distress syndrome requiring extracorporeal membrane oxygenation. The CCR5Δ32 variant was genotyped in all subjects.

**Results:**

The CCR5Δ32 allele was found in one single subject, who developed a very mild form and was not hospitalized.

**Conclusions:**

The CCR5Δ32 allele was not found to be associated with the risk of H1N1pdm09 infection or with a severe disease course.

## Background

The swine-origin H1N1 influenza A virus that appeared in Mexico in 2009 (H1N1pdm09) caused the first flu pandemic of the 21st century. H1N1pdm09 infection resulted in a substantial diseases burden with a minority of infected subjects developing life-threatening respiratory complications. Estimates of H1N1pdm09-associated mortality are similar in magnitude to that of seasonal influenza, but most deaths occurred in healthy people younger than 65 years old [1,2]. Also, strong differences in mortality were observed depending on ethnicity and geographic location [1,2]. Whereas these trends are likely to be partially explained by epidemiological aspects (e.g. previous exposure to antigenically similar viruses), host genetic and non-genetic factors were proposed to play a role. In particular, non-genetic risk factors were shown to include pregnancy, morbid obesity, cardiovascular disease, diabetes, and immunosuppression [2]. Nonetheless, a large majority of severely affected subjects were healthy and did not present any risk condition, suggesting that genetic effects might modulate H1N1pdm09 disease course.

Indeed, studies based on historical registries indicated that genetic factors affect the risk of death due to influenza virus infection [3]. Likewise, the familial aggregation of influenza A/H5N1 cases, the paucity of cases among highly exposed groups, and the occurrence of related cases separated in time and space led Horby and coworkers to indicate that host genetic factors play an important role in the susceptibility to this infection [4].

Clearly, the identification of host genetic factors that modulate the susceptibility to and the severity of influenza virus infection is of paramount importance to develop preventive strategies and therapeutic interventions.

Recently, a case–control study of 91 Mexican patients with confirmed severe pneumonia from H1N1pdm09 infection identified three independent association signals [5]. Nevertheless, no subsequent analysis addressed the role of these variants and the study design was likely to yield false positives, as noted [6]. Several other reports have explored the role of human genetic variants in the modulation of H1N1pdm09 infection severity, but only two variants have been analyzed in at least two independent studies. The first one, a functional variant in the *IFITM3* gene (rs12252), was originally described in a cohort of 53 influenza UK cases who developed severe respiratory complications [7]. The association was confirmed in a study of H1N1 cases in China, where the frequency of the minor predisposing genotype is much higher [8]. Nonetheless, the rarity of the risk genotype (only 3 homozygotes drove the association in the UK study) suggests that this variant makes a contribution to H1N1 disease severity in Europe. The second candidate variant is represented by a 32-bp polymophic deletion in the *CCR5* gene, the so-called CCR5Δ32 allele, which is almost exclusive to individuals with European ancestry. In a Canadian sample of 9 Caucasian patients with severe H1N1pdm09 infection Keynan et al. [9] found a nearly 5-fold enrichment of CCR5Δ32 heterozygotes compared to the expectation based on allele frequency in populations with European ancestry. This association found support by the description of a fatal case of H1N1pdm09 infection: the young patient was homozygous for the CCR5Δ32 allele, a status that occurs in a small minority of subjects (0.02% to 2% depending on the population) [10]. Also, studies in a *Ccr5*^*−/−*^ mice have indicated that, when infected with influenza A virus, these animals display accelerated macrophage accumulation in the lungs and increased mortality compared to wild-type mice [11].

Herein we investigated whether the CCR5Δ32 associates with disease severity in H1N1pdm09 infected southern European patients.

## Methods

### Patients

We analyzed 29 subjects with laboratory confirmed H1N1 infection who were referred to the Infectious Disease Unit of the San Gerardo Hospital, Monza, Italy, in winter 2009–2010. Most patients described herein were recruited immediately after the H1N1 outbreak in a relatively short time period. Peripheral blood samples were obtained from H1N1pdm09 infected patients as part of their care pathway. All patients signed an informed consent allowing leftover blood samples to be used for research purposes. The informed consent form was approved by the Internal Review Board of the San Gerardo Hospital and explicitly mentioned the use of leftover samples for genetic research. Samples were stored and processed anonymously. The authors have presently no access to identifying information and cannot relate genetic data to individual subjects. The study was conducted in accordance with the ethical standards of the Helsinki Declaration. All subjects were of European ancestry (27 Italians and one Spanish) with the exception of a Chinese patient (in the mild form group). The clinical characteristics of these subjects are summarized in Table 1.

**Table 1:** Clinical characteristics and CCR5Δ32 allelic status for the 29 H1N1pdm09 patients

**Flu form**	**Disease course**	**Number of subjects**	**Males/females**	**Self-reported ancestry (number)**	**Number of CCR5Δ32 heterozygotes**
Severe	ARSD requiring ECMO	7	6/1	Italian (6), Spanish (1)	0
Intermediate	Pulmonary disease requiring oxygen therapy	7	3/4	Italian (7)	0
Mild	No complications, only oxygen administration	7	3/4	Italian (6), Chinese (1)	0
Very mild	No hospitalization, no complications	8	4/4	Italian (8)	1

### Genotyping

**Figure 1:**
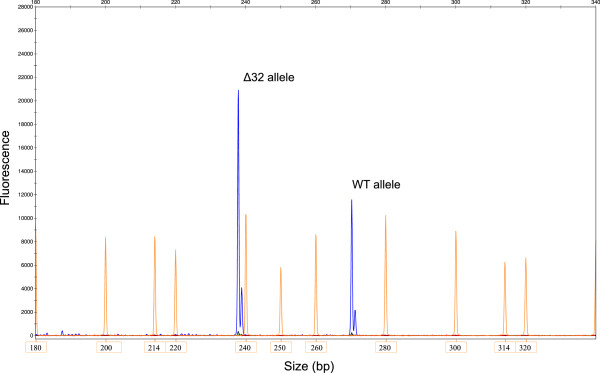
**GeneScan analysis of a subject heterozygous for the CCR5Δ32 allele.** Fluorescence-labelled PCR products (blue peaks) were separated by capillary electrophoresis with a size standard (red peaks).

For all subjects genomic DNA was obtained from peripheral blood mononuclear cells. The CCR5Δ32 variant was genotyped by PCR amplification using a forward primer (CCR5-D32-F: TTTACCAGATCTCAAAAAGAAG) and a fluorescently labelled reverse primer (CCR5-D32-R: GGAGAAGGACAATGTTGTAGG, FAM). PCR-amplified fluorescently tagged samples were run on 3500xL Genetic Analyzer (Life Technologies) using the GeneScanTM 600 LIZ® size standard (Life Technologies). The PCR amplicons were separated by size electrophoresis and the dye labeled products were identified by fluorescence detection. GeneMapper® Software Version 4.0 was applied to size the alleles (Figure 1).

## Results

We recruited 29 subjects with laboratory confirmed H1N1 infection. Twenty-one of these patients were hospitalized; seven of them developed acute respiratory distress syndrome (ARDS) and required extracorporeal membrane oxygenation (ECMO). These subjects are referred to as having a severe flu form (Table 1); two of them were overweight, one was diabetic, and one suffered from non-Hodgkin lymphoma. The patient suffering from non-Hodgkin lymphoma was not undergoing any therapy and was disease-free at the moment of hospitalization; the diabetic patient had his disease controlled through therapy. Seven other subjects developed pulmonary disease, required oxygen therapy administration alone and the disease course did not present life-threatening respiratory symptoms (intermediate gravity, Table 1). The seven remaining hospitalized patients only required oxygen therapy administration and the disease course presented no complications (mild form, Table 1). Finally, 8 additional infected subjects were not hospitalized (very mild form). All patients survived their illness. For all of them the polymorphic 32 bp deletion in the *CCR5* gene was genotyped.

The reported frequency of the CCR5Δ32 allele in Italy is 0.055 (0.098 in Spain) [12], resulting in an expected frequency of heterozygotes around 10%. Among the H1N1pdm09-infected subjects only one was found to carry the CCR5Δ32 allele: the patient developed a very mild form and was not hospitalized. Thus, the CCR5Δ32 allele was not found to be associated with the risk of H1N1pdm09 infection or with a severer disease course.

## Discussion

We evaluate the possible association between the CCR5Δ32 allele and severe H1N1pdm09 infection in patients form Southern Europe. CCR5 has been shown to be important for cell-mediated immune response against H1N1pdm09 [13,14] and the CCR5Δ32 allele confers increased susceptibility to symptomatic infection with other viral species such as West Nile virus (WNV) [15]. In this latter case, though, the association model is recessive - i.e. only homozygotes are at higher risk of developing a severe disease following WNV infection [15]. The recessive model is consistent with the loss-of-function effect of the 32-bp deletion in CCR5 and may apply to other phenotype associations, as it also describes a strong [16] but not complete [17] resistance to HIV-1 infection. The previously reported association between the CCR5Δ32 and severe H1N1pdm09 infection implied a non-recessive model, as a large excess of heterozygotes was described among critically ill patients [9]. Data herein do not confirm this finding and suggest that CCR5Δ32 does not play a major role in modulating H1N1pdm09 infection severity. It should be noted, however, that both Keynan’s [9] and this study suffer from the limitation of the small sample size and are underpowered to detect a recessive effect of CCR5Δ32 on H1N1 disease course. Also, the CCR5Δ32 allele tends to vary in frequency with a latitudinal cline [12], suggesting that population stratification might also affect the association results for this variant. Likewise, the rs12252 polymorphism in *IFITM3* previously associated with H1N1pdm09 disease severity shows strong frequency variation depending on ancestry. Its frequency is very low in Europe (but much higher in Asia) and a recent analysis of 34 severe H1N1pdm09 patients from the UK detected no association (whereas an effect of the rare homozygous genotype was detected for mild influenza) [18].

## Conclusions

Overall, results from these previous analyses together with those reported herein indicate that larger sample sizes will be required to perform robust association studies for H1N1pdm09 disease severity. Thus, provided that host genetics play a role in the susceptibility to this condition, most of its determinants in European ancestry subjects remain to be identified.

## Abbreviations

ARDS: Acute respiratory distress syndrome; ECMO: Extracorporeal membrane oxygenation.

## Competing interests

The authors declare that they have no competing interests.

## Authors’ contributions

MS, MC, and AG conceived and designed the study; RC and CP performed molecular genetic analyses; GM and MR recruited patients and provided clinical data; MS, MC, and AG wrote the manuscript. All authors read and approved the final manuscript.
